# Patient Demographics and Major Adverse Cardiovascular Events after Androgen Deprivation Therapy for Prostate Cancer

**DOI:** 10.1155/2024/2988289

**Published:** 2024-09-27

**Authors:** Christopher J. D. Wallis, Kevin C. Chen, Stuart Atkinson, Deborah M. Boldt-Houle

**Affiliations:** ^1^ Division of Urology Department of Surgery University of Toronto, 60 Murray Street, Koffler Ctr, 6th Floor, Toronto, Ontario M5G3L9, Canada; ^2^ Analytics and Information Xelay Acumen Group, Inc., 181 2^nd^ Ave, Suite 488, San Mateo, California 94401, USA; ^3^ Medical Affairs Tolmar Inc., 485 Half Day Road, Suite 400, Buffalo Grove, IL 60089, USA

## Abstract

**Background:**

The association between patient demographics and CV events after ADT using real-world data was evaluated. In addition to encompassing >30 times more patients than all previous MACE studies, this is the first study, to the best of our knowledge, to include a comprehensive listing of many demographic factors from one large, recent US dataset over a long period of time.

**Materials and Methods:**

The retrospective analysis of data in the Decision Resources Group (now Clarivate) Real World Evidence repository, representing >300M US patients from 1991 to 2020 across all US regions, was performed. Patients with PCa receiving ≥1 ADT injection were included. MACE risk after ADT initiation was evaluated for demographic and potential PCa-related risk factors. Kaplan–Meier survival curves were constructed, and Cox regression was used to evaluate the association between MACE risk and demographic/PCa-related risk factors.

**Results:**

Overall, MACE risk was slightly lower in the first year after ADT initiation (3.9%) vs. years 2–4 (∼5.2%). In a multivariate Cox model, MACE risk after ADT initiation was significantly higher for older vs. younger patients (adjusted HR per increasing year = 1.08, 95% CI: 1.07–1.09), men with a history of MACE vs. without (HR = 2.22, 95% CI: 1.72–2.88), men with very low BMI vs. normal or high BMI (HR for decreasing BMI per kg/m^2^ = 1.02, 95% CI: 1.01–1.03), White vs. Black patients (HR = 1.30, 95% CI: 1.08–1.55), and patients who did not use statins vs. those who did (HR = 1.13, 95% CI: 1.00–1.27). Of the PCa-related risk factors, MACE risk after ADT initiation was significantly higher for oncology vs. urology treatment setting (HR = 2.47, 95% CI: 2.12–2.88), patients with baseline metastasis vs. those without (HR = 2.30, 95% CI: 1.72–3.07), and patients treated with antagonists vs. agonists (HR = 1.62, 95% CI: 1.25–2.10).

**Conclusions:**

Demographic factors are important contributors to increased MACE risk for men with PCa on ADT. Clinicians should monitor risk factors and modify if possible.

## 1. Introduction

Androgen deprivation therapy (ADT) is the gold standard for ongoing treatment of advanced prostate cancer (PCa) [1]. While some studies have found associations between ADT and increased cardiovascular (CV) risk [2], this association is controversial [3, 4]. There is evidence that patients with PCa have higher CV risk than the general population, independent of ADT use, which is likely due to preexisting CV risk factors such as increasing age [5], lower body mass index (BMI) [6], race and ethnicity [7], a personal and/or family history of CV events [8, 9], tobacco use [10], and comorbidities (e.g., diabetes, hypercholesterolemia, and hypertension) [8]. Consequently, the European Society of Cardiology's Cardio-Oncology Guidelines recommend baseline CV risk assessment for PCa patients with preexisting CV disease, and annual CV reassessment during ongoing ADT [11]. In this study, we sought to evaluate the association between patient demographics and major adverse CV event (MACE) risk following ADT initiation using real-world data from patients with PCa. In addition to encompassing over 30 times more patients than all previous studies of MACE and the largest patient set of GnRH antagonist use in PCa with up to 4 years of longitudinal use, this is the first study, to the best of our knowledge, to include a comprehensive listing of many demographic factors from one large, recent US dataset over a long period of time (2010–2020).

## 2. Materials and Methods

### 2.1. Definitions

MACE was defined as myocardial infarction (MI), stroke, and all-cause death based on two recent studies in patients with PCa: HERO (randomized trial comparing relugolix and leuprolide over 48 weeks) [3] and PRONOUNCE (randomized trial comparing degarelix and leuprolide in patients with PCa and concomitant atherosclerotic CV disease over 12 months) [4].

### 2.2. Study Design

Data were collected from the Decision Resources Group (DRG, now called Clarivate) Real World Evidence repository, which links medical claims, prescription claims, and US Electronic Healthcare Records (EHR) to provide historical, longitudinal patient-level data. Other real-world studies have also used the DRG dataset [12, 13]. The DRG repository covered the majority of entities in the US healthcare system, including >300 million patients' medical and pharmacy claims and EHR data. We included patients with PCa who received at least one injection of ADT between 1991 and 2020. Exclusion criteria for the overall analysis set included patients who experienced a MACE within six months prior to ADT initiation. The exclusion of patients who experienced a MACE within six months prior to ADT initiation was chosen because patients are more likely to have a recurrence of MACE after their initial event and to align with the HERO trial [3]. We conducted the multivariable regression analyses both with and without the six-month exclusion criteria and found that the results were similar regardless of whether these patients were excluded. Here in this paper, we present the version with the six-month exclusion to match the HERO study methodology. The analyses of MACE risk by various demographic factors excluded patients who did not have data on the relevant demographic factor. Supplemental Tables 1–4 show the criteria used to identify study parameters.

### 2.3. Analysis Methodology

Continuous variables were presented as mean (standard deviation). The following demographic risk factors for MACE were evaluated using univariate Cox regression: increasing age, personal MACE history, body mass index (BMI <18.5, 18.5 to <25, 25 to <30, 30 to <35, and ≥35; BMI subgroups were based on the standard weight status associated with each BMI range [underweight, normal or healthy weight, overweight, obese, and severely obese, respectively] [14]), race (White vs. Black), statin use, ethnicity (Hispanic vs. non-Hispanic), family MACE history, hypertension, tobacco use, and diabetes. Additionally, the following PCa-related variables associated with increased MACE risk were evaluated using univariate Cox regression: treatment setting (oncology vs. urology), metastasis at baseline, ADT drug type (luteinizing hormone-releasing hormone (LHRH) agonist vs. gonadotropin-releasing hormone (GnRH) antagonist), increasing prostate-specific antigen (PSA), and ADT duration. Kaplan–Meier survival curves were constructed. All available variables were used in the univariate model. All risk factors with a significance of *p* < 0.05 in the univariate model were used in the multivariate Cox regression analysis to calculate the adjusted hazard ratio and 95% CI of each factor and identify significant MACE risk factors.

## 3. Results

### 3.1. Baseline Characteristics

The mean age was 73.7 (8.3) years. White and Black patients comprised 65.1% and 13.1% of the population, respectively, and 4.4% of patients were Hispanic (Table 1). Hypertension was the most common preexisting CV risk factor (78.0%), followed by diabetes mellitus (18.6%), tobacco use (10.0%), stroke (1.9%), heart failure (1.7%), and MI (1.4%). The mean BMI at baseline was 28.7 kg/m^2^. Metastatic disease was present at baseline in 2.6%, and a majority of patients were treated with an LHRH agonist (89.9%).

### 3.2. MACE Risk in All Patients

**Figure 1:**
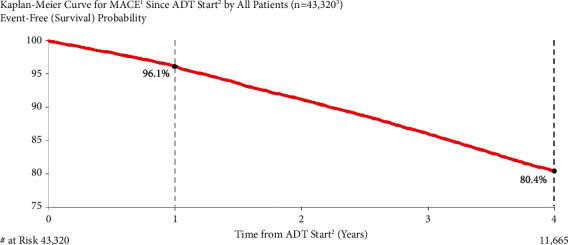
MACE risk in all patients. ^1^Major adverse cardiovascular events (recent urology studies HERO and PRONOUNCE definition) defined as myocardial infarction, stroke, and mortality from any cause. ^2^ADT = androgen deprivation therapy; date of the earliest LHRH injection recorded for patient. ^3^Excluded patients who had a MACE <6 months prior to ADT start.

The Kaplan–Meier estimate for MACE risk was 3.9% and 19.6% at one and four years following the start of ADT, respectively (Figure 1). Overall, MACE risk was slightly lower in the first year after ADT initiation compared to the subsequent years (3.9% in the first year versus ∼5.2% in years 2 through 4).

### 3.3. MACE Risk by Age

**Figure 2:**
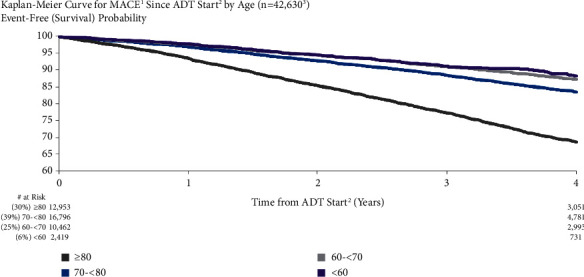
MACE risk for patients in different age subgroups. ^1^Major adverse cardiovascular events (recent urology studies HERO and PRONOUNCE definition) defined as myocardial infarction, stroke, and mortality from any cause. ^2^ADT = androgen deprivation therapy; date of the earliest LHRH injection recorded for patient. ^3^Patients who had a MACE <6 months prior to ADT start are excluded.

The risk of MACE was higher in older patients than in younger patients (Figure 2). One year after ADT start, MACE risk was 6.5%, 3.1%, 2.4%, and 2.2% in patients aged ≥80 years, 70 to <80 years, 60 to <70 years, and <60 years old, respectively. At four years after ADT initiation, MACE risk was 31.3%, 16.5%, 12.7%, and 11.7% for the same age groups, respectively. Both unadjusted and adjusted risks for MACE risk by increasing age (per year) were significant (unadjusted: HR = 1.07, 95% CI: 1.06–1.07; adjusted: HR = 1.08, 95% CI: 1.07–1.09).

### 3.4. MACE Risk by Treatment Setting

**Figure 3:**
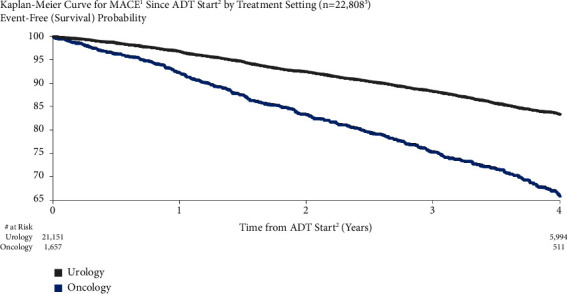
MACE risk by treatment setting. ^1^Major adverse cardiovascular events (recent urology studies HERO and PRONOUNCE definition) defined as myocardial infarction, stroke, and mortality from any cause. ^2^ADT = androgen deprivation therapy; date of the earliest LHRH injection recorded for patient. ^3^Excluded patients who had a MACE <6 months prior to ADT start and patients with unknown or both urology/oncology setting.

MACE risk was higher for patients treated in an oncology vs. urology setting (Figure 3). MACE risk was 7.9% vs. 3.3% for men treated in an oncology vs. urology setting at one year after ADT initiation and 34.0% vs. 16.5% at four years. Both unadjusted (HR = 2.22, 95% CI: 2.03–2.43) and adjusted MACE risks (HR = 2.47, 95% CI: 2.12–2.88) for men treated in an oncology vs. urology setting were significant.

### 3.5. MACE Risk by Baseline Metastasis

**Figure 4:**
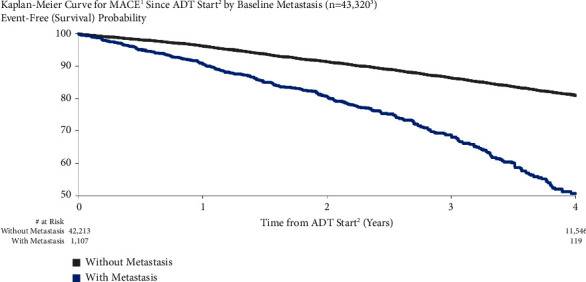
MACE risk by baseline metastasis. ^1^Major adverse cardiovascular events (recent urology studies HERO and PRONOUNCE definition) defined as myocardial infarction, stroke, and mortality from any cause. ^2^ADT = androgen deprivation therapy; date of the earliest LHRH injection recorded for patient. ^3^Patients who had a MACE <6 months prior to ADT start are excluded.

MACE risk was higher for patients with metastatic disease at baseline compared to those without 4.4% vs. 3.0% at one year after starting ADT and 22.5% vs. 17.3% at four years (Figure 4). Unadjusted (HR = 2.69, 95% CI: 2.40–3.03) and adjusted MACE risks (HR = 2.30, 95% CI: 1.72–3.07) for metastatic disease at baseline were significant.

### 3.6. MACE Risk by Personal MACE History

**Figure 5:**
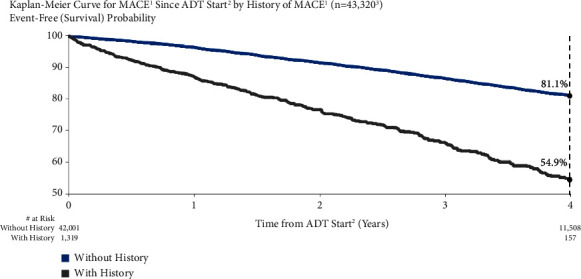
MACE risk by personal MACE history. ^1^Major adverse cardiovascular events (recent urology studies HERO and PRONOUNCE definition) defined as myocardial infarction, stroke, and mortality from any cause. ^2^ADT = androgen deprivation therapy; date of the earliest LHRH injection recorded for patient. ^3^Excluded patients who had a MACE <6 months prior to ADT start are excluded.

MACE risk was higher for men with a history of MACE compared to those without previous MACE (Figure 5). One year after starting ADT, MACE risk was 13.0% and 3.6% for men who had previously experienced a MACE and those who had not, respectively. At four years, MACE risk was 45.1% and 18.9%, respectively. Unadjusted and adjusted MACE risks for men with vs. without a history of MACE were significant: unadjusted HR = 2.76 (95% CI: 2.49–3.06) and adjusted HR = 2.22 (95% CI: 1.72–2.88).

### 3.7. MACE Risk by BMI

**Figure 6:**
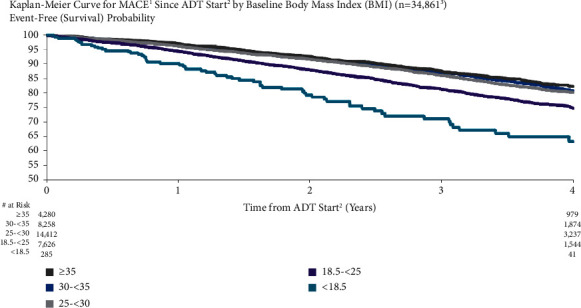
MACE risk for patients in different body mass index subgroups. ^1^Major adverse cardiovascular events (recent urology studies HERO and PRONOUNCE definition) defined as myocardial infarction, stroke, and mortality from any cause. ^2^ADT = androgen deprivation therapy; date of the earliest LHRH injection recorded for patient. ^3^Excluded patients who had a MACE <6 months prior to ADT start.

MACE risk was higher for patients with very low BMI compared to those with normal or high BMI (Figure 6). For patients with BMI ≥35, 30 to <35, 25 to <30, 18.5 to <25, and <18.5, MACE risk at one year after ADT start was 3.1%, 3.6%, 3.8%, 5.7%, and 9.9%, respectively. Four years after ADT initiation, MACE risk was 17.7%, 19.2%, 19.8%, 25.2%, and 36.7%, respectively. The unadjusted and adjusted HRs for decreasing BMI per kg/m^2^ were 1.03 (95% CI: 1.03–1.04) and 1.02 (95% CI: 1.01–1.03), respectively.

### 3.8. MACE Risk by ADT Type

**Figure 7:**
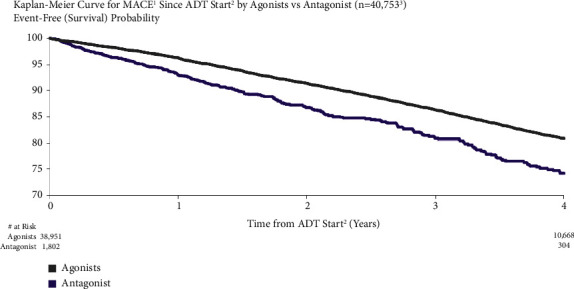
MACE risk by androgen deprivation therapy type. ^1^Major adverse cardiovascular events (recent urology studies HERO and PRONOUNCE definition) defined as myocardial infarction, stroke, and mortality from any cause. ^2^ADT = androgen deprivation therapy; date of the earliest LHRH injection recorded for patient. ^3^Excluded patients who have taken both an agonist and antagonist; patients who had a MACE <6 months prior to ADT start are excluded.

MACE risk was higher for patients treated with a GnRH antagonist vs. those treated with an LHRH agonist. For patients treated with GnRH antagonist vs. LHRH agonists, MACE risk was 7.0% vs. 3.8% at one year after ADT initiation and 25.8% vs. 19.1% at four years (Figure 7). Both the unadjusted and adjusted incidences of MACE were higher for men treated with GnRH antagonists compared to the LHRH agonists (unadjusted: HR = 1.54, 95% CI: 1.38–1.72 and adjusted: HR = 1.62, 95% CI: 1.25–2.10).

### 3.9. MACE Risk by Race

**Figure 8:**
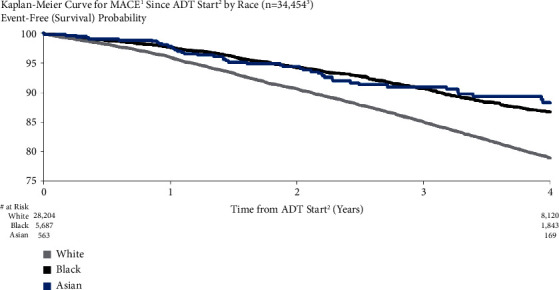
MACE risk by race. ^1^Major adverse cardiovascular events (recent urology studies HERO and PRONOUNCE definition) defined as MI, stroke, and mortality from any cause. ^2^ADT = androgen deprivation therapy; date of the earliest LHRH injection recorded for patient. ^3^Excluded patients who had a MACE <6 months prior to ADT start.

A comparison of race subgroups showed that MACE risk was higher for White patients than for Black and Asian patients (4.0%, 2.4%, and 2.2% for White, Black, and Asian patients, respectively, at one year after ADT initiation; 21.0%, 13.3%, and 11.7, respectively, at four years) (Figure 8). The unadjusted and adjusted HRs for White vs. Black patients were 1.68 (95% CI: 1.56–1.82) and 1.30 (95% CI: 1.08–1.55).

### 3.10. MACE Risk by Statin Use

**Figure 9:**
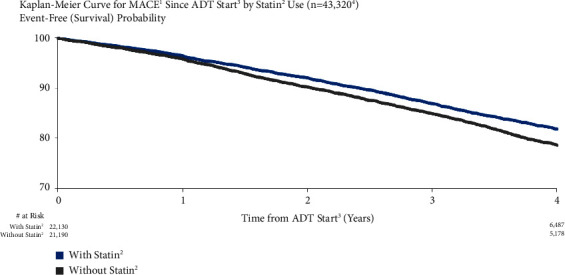
MACE risk by statin use. ^1^Major adverse cardiovascular events (recent urology studies HERO and PRONOUNCE definition) defined as myocardial infarction, stroke, and mortality from any cause. ^2^Patients who had taken statin medication (patients with events categorized as statins if the medication was dated prior to the first event after ADT start). ^3^Date of the earliest LHRH injection recorded for patient. ^4^Patients who had a MACE <6 months prior to ADT start are excluded.

The risk of MACE was higher for patients who did not use statins vs. those who did. MACE risk for patients who did not use statins vs. those who did was 3.8% vs. 4.0%, respectively, at one year after ADT start and 19.0% vs. 20.8% at four years (Figure 9). Both unadjusted (HR = 1.24, 95% CI: 1.18–1.30) and adjusted (HR = 1.13, 95% CI: 1.00–1.27) risks were significant.

### 3.11. MACE Risk by Ethnicity

Analysis by ethnicity found that MACE risk was higher for non-Hispanic than for Hispanic patients: 3.8% vs. 2.7% at one year and 19.7% vs. 13.3% at four years after ADT initiation (Supplemental Figure 1). The unadjusted MACE risk was significant (HR = 1.41, 95% CI: 1.24–1.62) but the adjusted risk was not (HR = 1.28, 95% CI: 0.88–1.87).

### 3.12. MACE Risk by Family MACE History

MACE risk was 4.2% vs. 3.9% for patients with and without family MACE history, respectively, at one year after starting ADT and 22.3% vs. 19.4% at four years (Supplemental Figure 2). The unadjusted risk was significant (HR = 1.14, 95% CI: 1.04–1.25), but the adjusted risk was not (HR = 1.15, 95% CI: 0.93–1.44).

### 3.13. MACE Risk by Hypertension

The risk of MACE was similar for patients with and without hypertension. The risk of MACE after initiating ADT for patients without and with hypertension was 4.0% vs. 3.4% at one year and 19.9% vs. 18.4% at four years (Supplemental Figure 3). The unadjusted risk was significant (HR = 1.07, 95% CI: 1.01–1.14), but the adjusted risk was not (HR = 1.04, 95% CI: 0.89–1.22).

### 3.14. MACE Risk by Tobacco Use

The risk of MACE was similar for patients with and without a history of tobacco use (Supplemental Figure 4). MACE risk was 4.1% vs. 3.8% at one year after ADT initiation for men with and without a history of tobacco use, respectively, and 19.6% vs. 20.0% at four years (HR = 1.03, 95% CI: 0.95–1.12).

### 3.15. MACE Risk by Diabetes

The risk of MACE was similar for patients with and without diabetes. The risk of MACE after initiating ADT for patients without and with diabetes was 3.8% vs. 3.9% at one year and 19.5% vs. 19.6% at four years (unadjusted HR = 1.03, 95% CI: 0.97–1.09) (Supplemental Figure 5).

### 3.16. Multivariate Analyses

**Figure 10:**
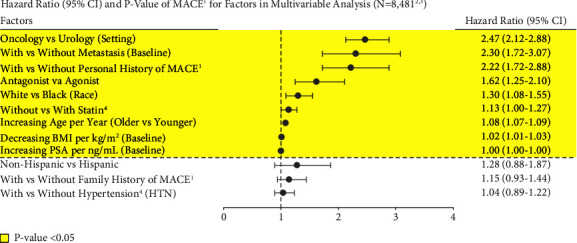
Forest plot of significant MACE risk factors. ^1^Major adverse cardiovascular events (recent urology studies HERO and PRONOUNCE definition) defined as myocardial infarction, stroke, and mortality from any cause. ^2^Excluded patients who had a MACE <6 months prior to ADT start; ADT = androgen deprivation therapy. ^3^Excluded patients who had taken both an agonist and antagonist; without body mass index (BMI), prostate-specific antigen (PSA), age, and ethnicity data; who were not of white or black race; and with unknown urology/oncology setting or patients who had both urology/oncology setting. ^4^Patients who had taken statin/hypertension medication or diagnosed with hypertension disease (patients with events categorized as statins/hypertension if the medication/diagnosis was dated prior to the first event after ADT start).

The factors with the greatest impact on MACE risk for men with PCa on ADT were, in descending order, increasing age (HR = 1.08 per year), decreasing BMI (HR = 1.02 per kg/m^2^), oncology vs. urology setting (HR = 2.47), baseline metastasis (HR = 2.30), personal MACE history (HR = 2.22), and treatment with an antagonist vs. agonist (HR = 1.62), *p* < 0.005 for all (Figure 10, Table 2).

## 4. Discussion

Although the ultimate goal of treating men with PCa using ADT is to prevent or slow disease progression by achieving castration levels of testosterone, monitoring and maintaining good CV health is also an important treatment consideration. The link between ADT and CV mortality is likely multifactorial, driven by the high baseline CV risk factor burden in the PCa population, the role of testosterone [15] and potentially follicle-stimulating hormone on CV risk [16], and ADT effects including increased large artery stiffness [17]. Additionally, common risk factors were increasing age [5], current or previous tobacco use [18], hypertension [18, 19], and physical frailty [19]. Therefore, it is likely that a large part of the increased CV risk observed after ADT initiation stems from preexisting CV risk factors.

Evidence indicates a significant presence of underassessed and undertreated CV risk factors in this patient group. In a comprehensive analysis of 90,494 men with PCa, 68% underwent CV risk assessment [20]. Among them, 54% exhibited uncontrolled CV risk factors, with 30% not receiving the appropriate medication [20]. These findings highlight a potential explanation for increased CV risk in patients with PCa.

Some of our findings reinforced the previously published literature and add to the body of knowledge on MACE risk by rigorously and methodically quantifying the impact of each risk factor in this large and recent US database. Overall, MACE risk at one year was similar to previously published data (4% vs. 3–6% in HERO [3] and 4–6% in PRONOUNCE [4]). Consistent with the literature that increasing age is an independent CV risk factor [21], MACE risk was higher for older patients. In fact, at 4 years after starting ADT, MACE risk was almost 3 times higher in patients aged ≥80 years (31%) vs. <60 years (12%). This may be due to the increased prevalence of comorbidities that contribute to CVD, such as diabetes [22], obesity [23], and frailty [24]. The mechanisms by which certain demographics influence MACE risk are multifaceted. Aging leads to CV changes such as increased arterial stiffness, left ventricular hypertrophy, and electrical dysfunction such as arrhythmias, which are key MACE risk factors [21]. Older adults also face endothelial dysfunction and a proinflammatory state, both promoting atherosclerosis and thrombotic events. Comorbidities such as diabetes and obesity also critically influence MACE risk. Diabetes accelerates atherosclerosis and causes microvascular complications [22], while obesity is associated with hypertension, dyslipidemia, insulin resistance, and inflammation [23]. Frailty, characterized by decreased physiological reserves and multiple comorbidities, heightens vulnerability to cardiovascular events [24]. MACE risk was twice as high for patients treated in an oncology vs. urology setting (34 vs. 17% at 4 years after ADT initiation), likely due to sicker population (i.e., more advanced prostate cancer disease and/or the potential presence of other concurrent or subsequent cancers, which may carry inherent CV risks) and receive additional therapies that may pose additional CV risks. Our analysis is consistent with a previous study that reported a higher likelihood of death during follow-up, more comorbid illnesses, and greater use of chemotherapy among patients treated by oncologists vs. urologists [25], given that patients treated by oncologists likely have more advanced disease. Patients with more advanced PCa (presence of baseline metastasis) and higher baseline PSA had higher MACE risk compared to patients without baseline metastasis and lower PSA. This is consistent with the finding that thromboembolic disease risk in men with PCa treated with endocrine therapy was higher for men with metastatic disease [26]. In patients with baseline metastasis and higher PSA, the presence of advanced cancer burden may lead to systemic inflammation, endothelial dysfunction, and hypercoagulability, all of which are established factors contributing to CV risk. Also consistent with other publications is the finding that MACE risk is higher in men who previously experienced a MACE [9, 27], over 2 times greater at 4 years after starting ADT than men without MACE history (45 vs. 19%). Potential factors contributing to this elevated risk in individuals with a prior MACE include the persistence of underlying CV conditions and ongoing vascular damage [9, 27].

Importantly, many of our findings shed new light on controversies in the previous literature and contradict conventional perceptions and therefore are useful in updating current clinical hypotheses. Contrary to popular belief, very low BMI was associated with higher MACE risk (37 vs. 18% for BMI <18.5 vs. ≥35 at 4 years after starting ADT) [28]. A potential explanation for increased risk in underweight patients is cancer cachexia [29, 30], a secondary disease in cancer patients, leading to significant weight loss, reduced skeletal muscle mass, and depletion of fat and heart muscle. Individuals with very low BMI, often due to cachexia, face higher MACE risk due to severe nutritional deficiencies and muscle loss [29, 30]. This impaired nutritional status leads to disrupted glucose metabolism and increased insulin resistance. Additionally, chronic inflammatory conditions common in these individuals further elevate the risk of MACE [31, 32]. Contrary to the previous literature [3], MACE risk was higher for patients treated with antagonists vs. agonists. A potential explanation is the selection bias by healthcare providers, who may be more likely to prescribe a GnRH antagonist to patients with a history of CV events or patients whose disease is more advanced since physicians want to avoid the clinical flare secondary to testosterone surge induced by agonists. However, higher MACE risk for the antagonist group remained even after adjustment for baseline demographic differences, including metastasis and baseline PSA. Contrary to previous findings, MACE risk was higher for White vs. Black patients with PCa. This could be due to the survival bias of Black patients with PCa as Black patients may have passed away from their PCa before MACE occurred. Interestingly, MACE risk in this analysis was higher for patients who did not use statins, potentially because statins may have known pleiotropic effects such as reducing the ischemic burden of the myocardium and preventing coronary occlusion [33]. In a separate but potentially synergistic effect, statins may decrease both advanced PCa risk [34] and PCa mortality risk [35]. Higher MACE risk in patients who did not use statins may also reflect undiagnosed hypercholesterolemia. In contrast to previous literature [36], increasing the ADT duration was not associated with higher MACE risk. It should be noted that patients excluded from the multivariable analysis due to missing data from one or more of the confounding variables had more MACE and similar, or potentially fewer, comorbidities than patients who were included in the multivariable analysis. In fact, MACE risk was slightly higher for the excluded population vs the included population (HR = 1.12, 95% CI: 1.05–1.19, *p*=0.001). These findings indicate that the population included in the multivariable analysis did have a disproportionately higher incidence of MACE.

Our study had limitations. Our findings rely on a database using ICD codes, whose reliability is constrained by the coding accuracy [37, 38]. Any misclassification bias is likely uniform across subgroups. Additionally, retrospective studies are hypothesis generating rather than confirmatory. The large size (∼45,000 patients from a database containing >300 million patients), extensive time period (2010–2020), and diversity of the dataset give weight to the results being an accurate representation of the current clinical experience. Despite potential incomplete data from the retrospective design, the large analysis population minimizes the missing data impact. Given that some of our findings conflict with the results from previously published studies, a large prospective trial with adjudicated CV endpoints (i.e., RADICAL PC2 and REPLACE CV [39, 40]) would provide valuable data. Another limitation of retrospective studies is that, despite rigorous statistical methods, residual confounding may persist—this risk is common in all retrospective analyses. Finally, standard PCa treatment regimens may have evolved over the study period, as seen in other long-term retrospective analyses. While our study identified demographic factors associated with MACE risk, other important residual confounding factors that may impact CV health (i.e., genetic markers, lifestyle and dietary habits, psychological factors, environmental exposure, and socioeconomic status) were not in the database and thus could not be adjusted. Future research should assess these factors to enhance our understanding of MACE risk factors in this population. This study has multiple comparisons that lead to a risk of type I errors. We initially performed univariable analyses on 15 factors to identify those significantly associated with MACE. Twelve factors were found to be significant and were subsequently included in the multivariable regression model. While this approach helped to reduce the number of variables and comparisons in the multivariable model, it does not fully address the risk of type 1 errors from multiple comparisons. Future analyses could incorporate correction methods, such as the Bonferroni correction or false discovery rate adjustments, to more robustly control for multiple comparisons.

Men with PCa face a higher risk of dying from CV disease than cancer itself [41]. Effective CV management can significantly enhance the overall survival. This study identifies key demographic factors impacting MACE, aiding clinicians in monitoring and treating CV risk factors. A multidisciplinary care team (e.g., primary care physician, urologist, oncologist, and cardiologist/cardio-oncologist) should collaborate for optimal CV treatment. If resources are lacking, a primary care provider should lead CV monitoring. These data support the importance of multidisciplinary care for these men as primary care physicians may be better positioned to provide the supportive care and risk mitigation necessary to improve cardiovascular outcomes for men receiving ADT.

## 5. Conclusions

Overall, MACE risk after ADT initiation increased consistently over the first four years (∼5% per year). The most significant risk factors for MACE included increasing age, oncology vs. urology setting, baseline metastasis, MACE history, and treatment with an antagonist vs. agonist. These results indicate that demographic factors are important contributors to increased MACE risk for men with PCa treated with ADT. Although MACE risk factors may be generally known to clinicians prescribing ADT, our analyses quantified the impact of each MACE risk factor, thus reinforcing the importance of monitoring patients with PCa and modifying MACE risk factors if possible. Appropriate management of CV health, including MACE risk factors, may have a greater impact on patients' quality of life and survival than the PCa.

## Figures and Tables

**Table 1:** Baseline demographics of patients who received at least one injection of androgen deprivation therapy.

Categories		All patients *N* = 43,320	MACE^1^*N* = 7,275	No MACE^1^*N* = 36,045
Age at the time of starting ADT^2^—year	Mean (SD)	73.7 (8.3)	76.4 (7.6)	73.2 (8.3)

Race	White, %	65.1	74.0	63.3
	Black, %	13.1	9.8	13.8
	Asian, %	1.3	0.9	1.4
	Other, %	2.3	1.8	2.4

Ethnicity	Hispanic, %	4.4	3.1	4.7
	Non-Hispanic, %	76.9	80.9	76.1

Prostate cancer	Metastatic baseline, %	2.6	4.1	2.2
	Metastatic all-time, %	5.3	9.7	4.4

Treatment regiment	LHRH^3^ agonist, %	89.9	88.6	90.2
	GnRH^4^ antagonist, %	4.2	4.4	4.1

Pre-ADT^2^ comorbidities	>6 months pre-ADT^2^	3.0	5.2	2.6
	MACE history, %			
	Diabetes mellitus, %	18.6	20.8	18.2
	Hypertension, %	78.0	82.6	77.1
	Tobacco use, %	10.0	10.1	10.0
	No tobacco use, %	55.7	61.6	54.5
	Stroke, %	1.9	3.0	1.7
	Heart failure, %	1.7	2.5	1.5
	Myocardial infarction, %	1.4	2.5	1.2
	Baseline BMI^5^, kg/m^2^	28.7	28.2	28.8

Year of first treatment	1991–2000, %	0.0	0.0	0.0
	2001–2010, %	1.7	3.1	1.5
	2011−2010, %	98.2	96.9	98.5

^1^Major adverse cardiovascular events (recent urology studies HERO and PRONOUNCE definition) defined as MI, stroke, and mortality from any cause; patients who had a MACE <6 months prior to ADT start are excluded. ^2^ADT = androgen deprivation therapy. ^3^LHRH = luteinizing hormone-releasing hormone. ^4^GnRH = gonadotropin-releasing hormone. ^5^BMI = body mass index.

**Table 2:** Univariate and multivariate analysis results.

Factors	Univariate (*N* = 43,320^1,2^)		Multivariate (*N* = 8,481^1,3^)	
	HR^4^ (95% CI)	*p* value	HR^4^ (95% CI)	*p* value
Oncology vs urology (setting)	2.22 (2.03–2.43)	<0.001	2.47 (2.12–2.88)	<0.001
With vs without metastasis (baseline)	2.69 (2.40–3.03)	<0.001	2.30 (1.72–3.07)	<0.001
With vs without personal history of MACE^5^	2.76 (2.49–3.06)	<0.001	2.22 (1.72–2.88)	<0.001
Antagonist vs agonist	1.54 (1.38–1.72)	<0.001	1.62 (1.25–2.10)	<0.001
White vs Black (race)	1.68 (1.56–1.82)	<0.001	1.30 (1.08–1.55)	<0.005
Without vs with statin^6^	1.24 (1.18–1.30)	<0.001	1.13 (1.00–1.27)	0.044
Increasing age per year (older vs younger)	1.07 (1.06–1.07)	<0.001	1.08 (1.07–1.09)	<0.001
Decreasing BMI^7^ per kg/m^2^ (baseline)	1.03 (1.03–1.04)	<0.001	1.02 (1.01–1.03)	<0.005
Increasing PSA^8^ per ng/mL (baseline)	1.00 (1.00–1.00)	<0.001	1.00 (1.00–1.00)	<0.001
Non-Hispanic vs Hispanic	1.41 (1.24–1.62)	<0.001	1.28 (0.88–1.87)	0.2
With vs without family history of MACE^1^	1.14 (1.04–1.25)	0.006	1.15 (0.93–1.44)	0.2
With vs without hypertension^6^ (HTN)	1.07 (1.01–1.14)	0.03	1.04 (0.89–1.22)	0.6
With vs without tobacco history	1.03 (0.95–1.12)	0.4	NA	NA
Without vs with diabetes^6^ (DIA)	1.03 (0.97–1.09)	0.4	NA	NA
Increasing ADT^9^ exposure per year	1.00 (0.99–1.01)	0.7	NA	NA

^1^Excluded patients who had a MACE <6 months prior to ADT start. ^2^Largest *N* out of all factors is shown for univariate analysis; Ns for each factor varies. ^3^Excluded patients who had taken both an agonist and antagonist; without BMI, age, and ethnicity data; who were not of white or black race; and with unknown urology/oncology setting or patients who had both urology/oncology setting. ^4^HR = hazard ratio; HR is a statistical measurement of how often a particular event happens in one group compared to how often it happens in another group, over time. ^5^Major adverse cardiovascular events (recent urology studies HERO and PRONOUNCE definition) defined as myocardial infarction, stroke, and mortality from any cause. ^6^Patients who had taken statin/hypertension/diabetes medication or diagnosed with hypertension/diabetes disease (patients with events categorized as statins/hypertension/diabetes if the medication/diagnosis was dated prior to the first event after ADT start). ^7^BMI = body mass index. ^8^PSA = prostate-specific antigen. ^9^ADT = androgen deprivation therapy.

## Data Availability

The data that support the findings of this study are available from the corresponding author upon reasonable request. Restrictions apply to the availability of data generated or analyzed during this study to preserve patient confidentiality or because they were used under license. The corresponding author (christopher.wallis@sinaihealth.ca) will on request detail the restrictions and any conditions under which access to some data may be provided.

## Supplementary Materials

Supplementary Materials Supplementary Figure 1: MACE risk by ethnicity. Supplementary Figure 2: MACE risk by family MACE history. Supplementary Figure 3: MACE risk by hypertension. Supplementary Figure 4: MACE risk by tobacco use. Supplementary Figure 5: MACE risk by diabetes. Supplementary Table 1: prostate cancer inclusion criteria by DRG. Supplementary Table 2: keywords to extract ADT data. Supplementary Table 3.1: keywords used to extract CV events. Supplementary Table 3.2: ICD codes used to extract CV events. Supplementary Table 4.1: comorbidity inclusion criteria–diabetes. Supplementary Table 4.2: comorbidity inclusion criteria–hypertension. Supplementary Table 4.3: comorbidity inclusion criteria–hypercholesterolemia.
