# Supplementary material for: Patient Demographics and Major Adverse Cardiovascular Events after Androgen Deprivation Therapy for Prostate Cancer
Source: Adv Urol. 2024 Sep 27;2024:2988289. doi: 10.1155/2024/2988289 (PMC11452244; doi:10.1155/2024/2988289)
Supplement: Supplementary Materials — Supplementary Figure 1: MACE risk by ethnicity. Supplementary Figure 2: MACE risk by family MACE history. Supplementary Figure 3: MACE risk by hypertension. Supplementary Figure 4: MACE risk by tobacco use. Supplementary Figure 5: MACE risk by diabetes. Supplementary Table 1: prostate cancer inclusion criteria by DRG. Supplementary Table 2: keywords to extract ADT data. Supplementary Table 3.1: keywords used to extract CV events. Supplementary Table 3.2: ICD codes used to extract CV events. Supplementary Table 4.1: comorbidity inclusion criteria–diabetes. Supplementary Table 4.2: comorbidity inclusion criteria–hypertension. Supplementary Table 4.3: comorbidity inclusion criteria–hypercholesterolemia. [file 2988289.f1.docx]

**Supplementary Materials**

**Figure 1. MACE Risk by Ethnicity**

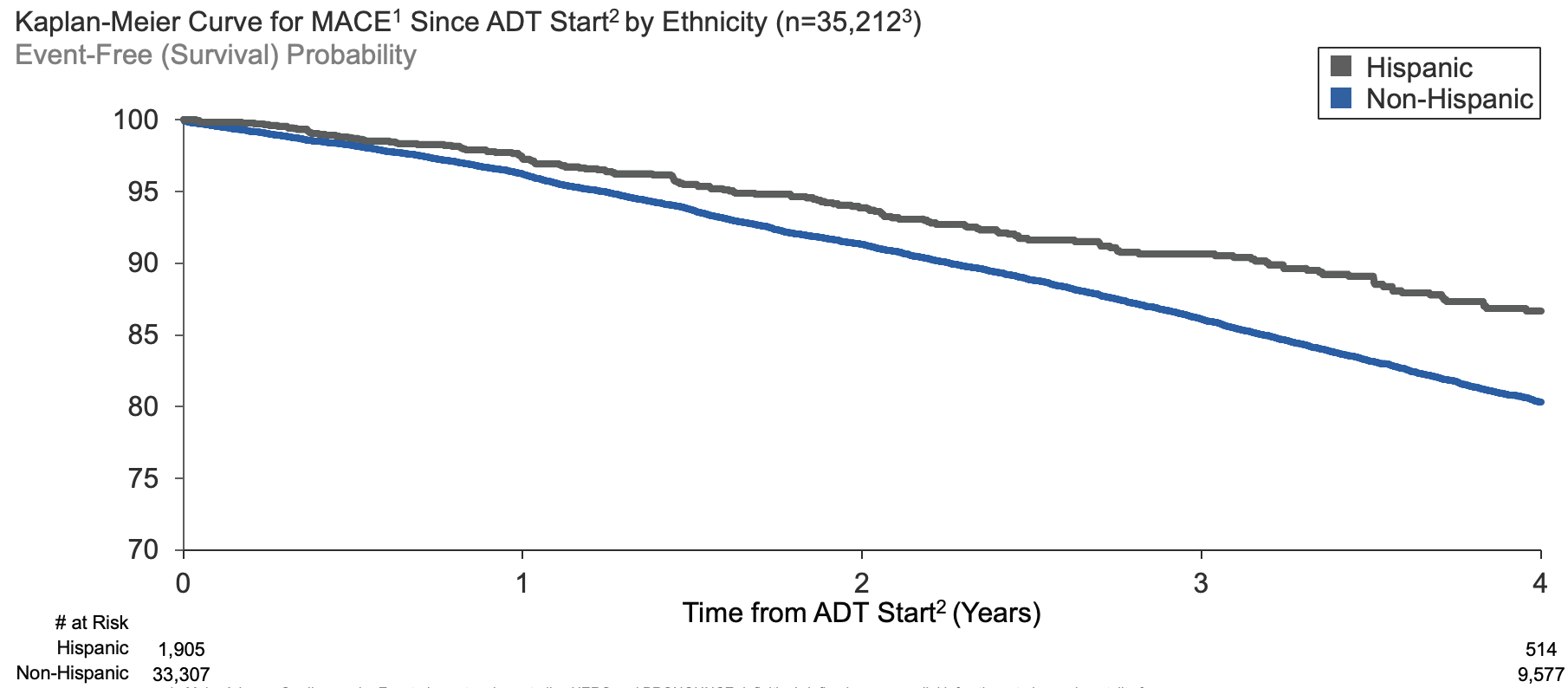


1 Major Adverse Cardiovascular Events (recent urology studies HERO and PRONOUNCE definition) defined as myocardial infarction, stroke, and mortality from any cause

2 ADT=Androgen deprivation therapy; Date of earliest LHRH injection recorded for patient

3 Excluded patients who had a MACE <6 months prior to ADT Start and patients with no ethnicity data

**Figure 2. MACE Risk by Family MACE History**


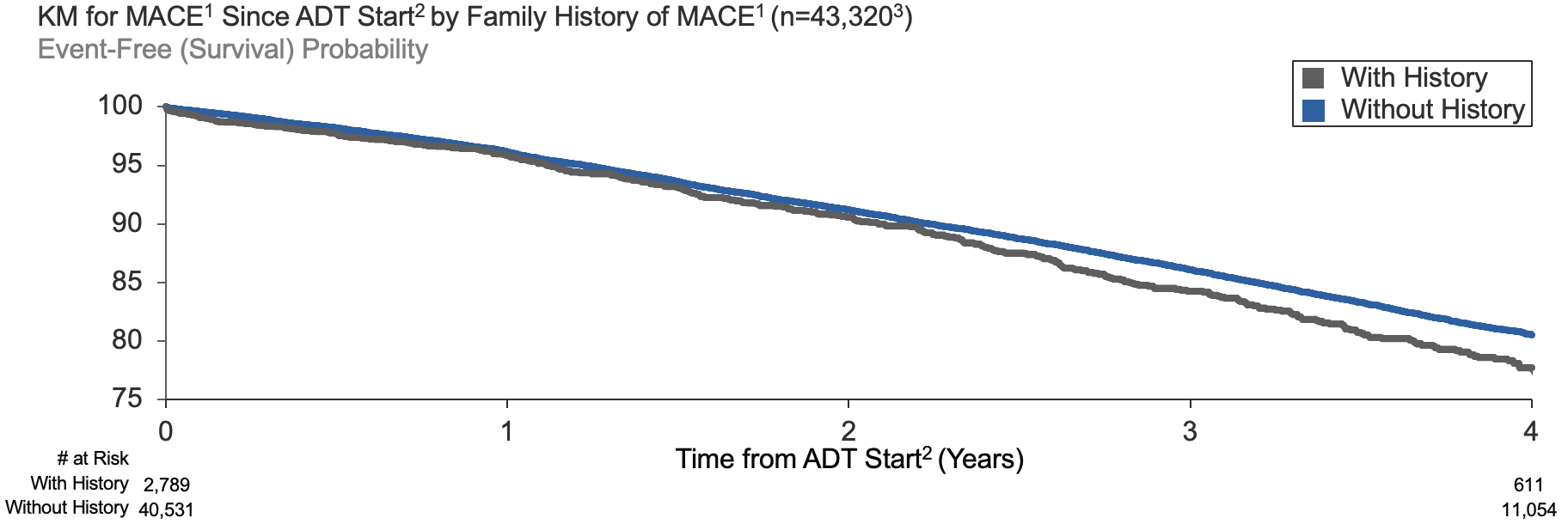


1 Major Adverse Cardiovascular Events (recent urology studies HERO and PRONOUNCE definition) defined as myocardial infarction, stroke, and mortality from any cause

2 Date of earliest LHRH injection recorded for patient

3 Excluded patients who had a MACE <6 months prior to ADT Start are excluded

**Figure 3. MACE Risk by Hypertension**

**
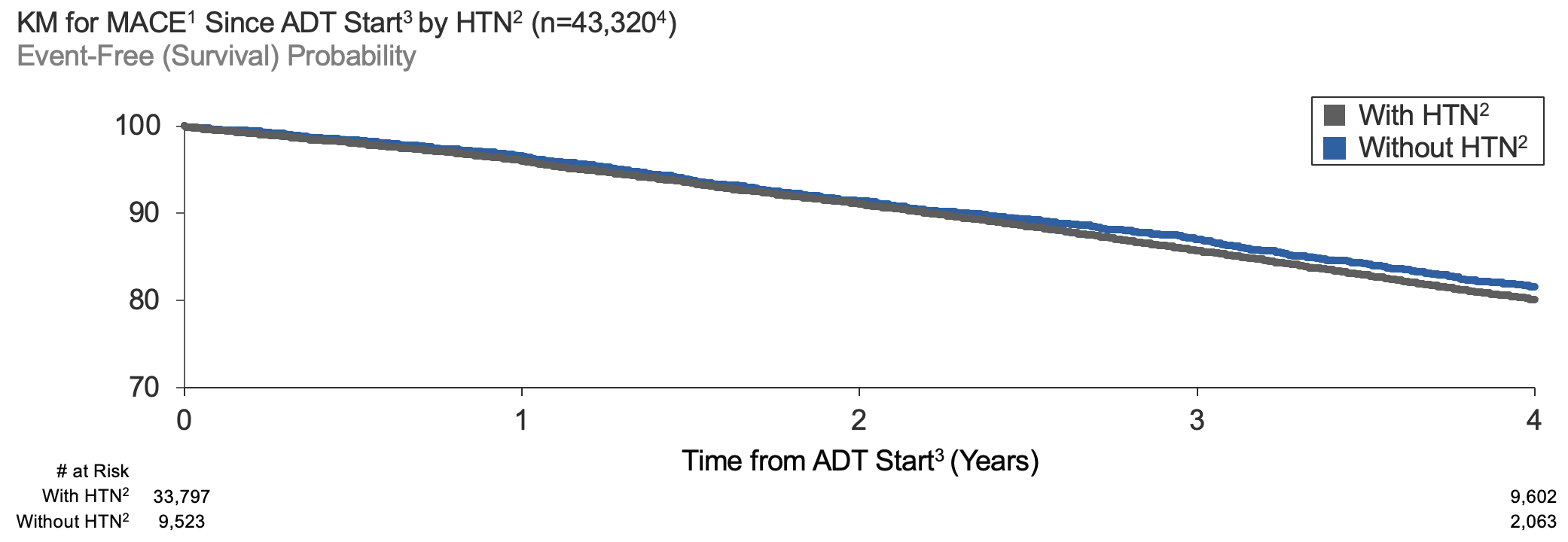
**

1 Major Adverse Cardiovascular Events (recent urology studies URO and PRONOUNCE definition) defined as MI, stroke, and mortality from any cause

2 Has taken hypertension medication or diagnosed with hypertension (patients with events categorized as hypertension if the medication/diagnosis was dated prior to the first event after ADT Start)

3 Date of earliest LHRH injection recorded for patient

4 Excluded patients who had a MACE <6 months prior to ADT Start

**Figure 4. MACE Risk by Tobacco Use**

**
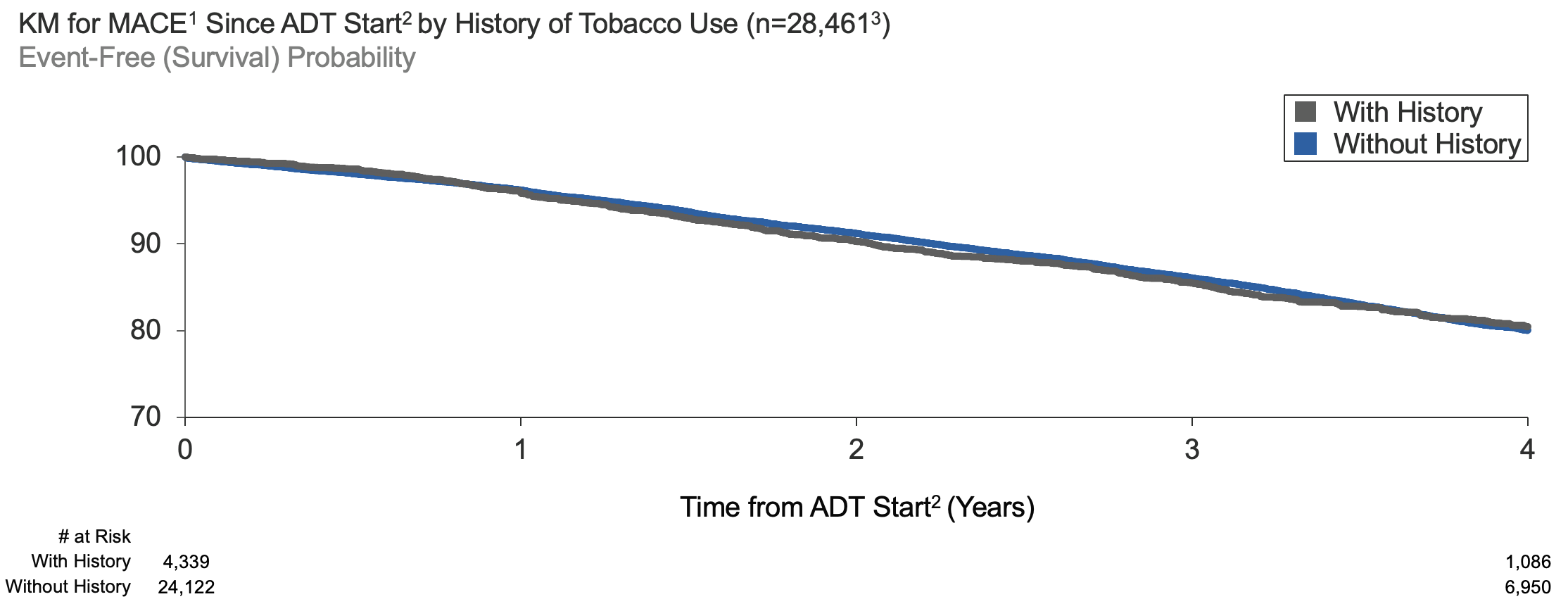
**

1 Major Adverse Cardiovascular Events (recent urology studies HERO and PRONOUNCE definition) defined as myocardial infarction, stroke, and mortality from any cause

2 Date of earliest LHRH injection recorded for patient

3 Patients who had a MACE <6 months prior to ADT Start are excluded

**Figure 5. MACE Risk by Diabetes**

**
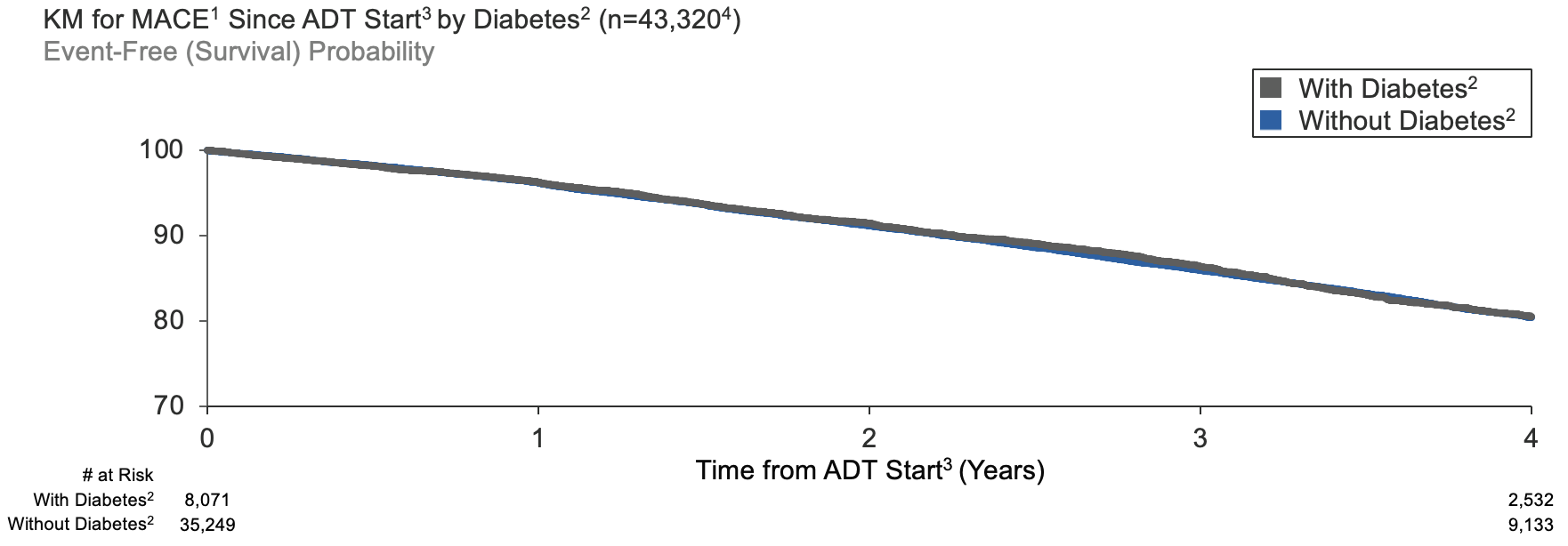
**

1 Major Adverse Cardiovascular Events (recent urology studies HERO and PRONOUNCE definition) defined as myocardial infarction, stroke, and mortality from any cause

2 Has taken diabetes medication or diagnosed with diabetes disease (patients with events categorized as diabetes if the medication/diagnosis was dated prior to the first event after ADT Start)

3 Date of earliest LHRH injection recorded for patient

4 Excluded patients who had a MACE <6 months prior to ADT Start

**Table 1. Prostate Cancer Inclusion Criteria by DRG**

| **Prostate Cancer Type** | **ICD-9** | **ICD-10** |
| --- | --- | --- |
| Malignant neoplasm of prostate | 185 | C61 |
| Secondary malignant neoplasm of genital organs | 198.82 | C79.82 |
| Carcinoma *in situ* of prostate | 233.4 | D07.5 |

**Table 2. Keywords to Extract ADT Data**

| **Generic Name(s)** | **Brand Name(s)** |
| --- | --- |
| Leuprolide | ELIGARD, LUPRON |
| Goserelin | ZOLADEX |
| Triptorelin | TRELSTAR |
| Degarelix | FIRMAGON |
| Histrelin | VANTAS, SUPPRELIN |

**Table 3.1. Keywords Used to Extract CV Events**

| **Keywords** |
| --- |
| AMI, Arrhythmia, Atrial, Attack, Cardiac, Cardiovascular, Cerebral, CV (CVD), Fibrillation, Heart, HF, Infarction, Ischemic, MACE, MI, Myocardial, Stroke, Tachycardia, Vascular, Ventricular |

**Table 3.2. ICD Codes Used to Extract CV Events**

| **CV Event** | **ICD-9** | **ICD-10** |
| --- | --- | --- |
| Myocardial Infarction | 410, 412 | I21, I22, I23, I25.2 |
| Stroke | 423, 430, 431, 432, 433, 434, 435, 436, 437 | G45, I64, I65, I66 |
| Unstable Angina | NA | I200 |
| Arterial Disease/Peripheral Vascular Disease | 17.56, 39.25, 39.26, 39.29, 39.5, 39.9, 411, 413, 414 | I24.0 |
| Heart Failure | NA | I09.9, I11, I13.0, I13.2, I25.5, I42.0, I42.5, I42.6, I42.7, I42.8, I42.9, I43, I50, I97.1, K76.1, P29.0 |

**Table 4.1. Comorbidity Inclusion Criteria – Diabetes**

| **Code Description** | **Code(s) or Keyword(s)** |
| --- | --- |
| Diagnosis Keywords | Diabetes, Diabetes Insipidus, Type 1 Diabetes, Type 2 Diabetes |
| Medication Keywords | Acarbose, ActoPlus Met, Actos, Adlyxin, Admelog, Afrezza, Albiglutide, Alogliptin, Amaryl, Apidra, Avandamet, Avandaryl, Avandia, Basaglar, Bromocriptine, Bydureon, Bydureon Bcise, Byetta, Canagliflozin, Chlorpropamide, Colesevelam, Cycloset, Dapagliflozin, Diabeta, Diabinese, Duetact, Dulaglutide, Empagliflozin, Ertugliflozin, Exenatide, Farxiga, Fiasp, Fortamet, Glimepiride, Glipizide, Glipizide XL, Glucophage, Glucophage XR, Glucotrol, Glucotrol XL, Glucovance, Glumetza, Glyburide, Glycron, Glynase, Glynase Prestab, Glyset, Glyxambi, Humalog, Humalog Kwikpen, Humulin, Humulin 70 / 30, Humulin n, Humulin r, Humulin r u-500, Humulin r u-500 kwikpen, Insulin Aspart, Insulin Degludec, Insulin Detemir, Insulin Glargine, Insulin Glulisine, Insulin Isophane, Insulin Lispro, Insulin Regular, Invokamet, Invokamet XR, Invokana, Janumet, Janumet XR, Januvia, Jardiance, Jentadueto, Kazano, Kombiglyze, Kombiglyze XR, Kwikpen, Lantus, Lantus Solostar, Levemir, Linagliptin, Liraglutide, Lixisenatide, Lyumjev, Metaglip, Metformin, Miglitol, Myxredlin, Nateglinide, Nesina, Novolin, Novolin n, Novolin r, Novolog, Novolog Flexpen, Novolog Penfill, Omeprazole, Onglyza, Oseni, Ozempic, Pioglitazone, Pramlintide, pramlintide acetate, PrandiMet, Prandin, Precose, Qtern, Repaglinide, Riomet, Rosiglitazone, Rxnx, Rybelsus, Saxagliptin, Segluromet, Semaglutide, Semglee, Sitagliptin, Soliqua, Starlix, Steglatro, Steglujan, Symlin, SymlinPen, SymlinPen 120, SymlinPen 60, Tanzeum, Tolazamide, Tolbutamide, Toujeo Max Solostar, Toujeo Solostar, Tradjenta, Tresiba, Trulicity, Victoza, Welchol, Xigduo XR |
| ICD-9 | 249, 250 |
| ICD-10 | E08, E09, E10, E11, E12, E13 |

**Table 4.2. Comorbidity Inclusion Criteria – Hypertension**

| **Code Description** | **Code(s) or Keyword(s)** |
| --- | --- |
| Diagnosis Keywords | Hypertension, HTN, Hypertensive, High Blood Pressure |
| Medication Keywords | Aartia, Accupril, Accuretic, Acebutolol, Aceon, Adalat CC, Afeditab, Aldactazide, Aldactone, Aldoril, Aliskiren, Altace, Amiloride, Amlobenz, Amlodipine, Apresoline, Aquazide, Atacand, Atenolol, Atorvastatin, Avalide, Avapro, Azilsartan, Azor, Benazepril, Bendroflumethiazide, Benicar, Betaxolol, Bisoprolol, Brevibloc, Bystolic, Caduet, Calan, Candesartan, Capoten, Captopril, Cardene, Cardizem, Cardura, Carospir, Cartia, Carvedilol, Catapres, Chlorothiazide, Chlorthalidone, Clevidipine, Cleviprex, Clonidine, Conjupri, Consensi, Coreg, Corgard, Corzide, Covera HS, Cozaar, Demadex, Dibenzyline, Dilacor XR, Dilt, Diltia, Diltiazem, Diltzac, Diovan, Diovan HCT, Diuril, Diuril Sodium, Doxazosin, Dutoprol, Dyazide, DynaCirc CR, Edarbi, Edarbyclor, Enalapril, Enalaprilat, Enduron, Epaned, Eplerenone, Eprosartan, Esidrix, Esmolol, Exforge, Exforge HCT, Felodipine, Fosinopril, Furosemide, Guanabenz, Guanfacine, Hydralazine, Hydrochlorothiazide, Hydroflumethiazide, Hytrin, Hyzaar, Indapamide, Inderal, Inderal LA, Innopran, Inspra, Irbesartan, Isoptin SR, Isradipine, Kapspargo Sprinkle, Katerzia, Labetalol, Lasix, Levamlodipine, Levatol, Lisinopril, Lopressor, Losartan, Lotensin, Lotrel, Maleate, Matzim, Mavik, Maxzide, Mecamylamine, Methyclothiazide, Methyldopa, Metolazone, Metoprolol, Micardis, Microzide, Midamor, Minipress, Minitran, Minoxidil, Moexipril, Monopril, Nadolol, Naturetin, Nebivolol, Nicardipine, Nifedipine, Nimodipine, Nisoldipine, Nitro TD Patch-A, Nitro-Bid, Nitro-Dur, Nitro-Time, Nitroglycerin, Nitrolingual Pumpspray, Nitromist, Nitrostat, Norvasc, Olmesartan, Oretic, Penbutolol, Perindopril, Phenoxybenzamine, Pindolol, Plendil, Prazosin, Prestalia, Prinivil, Procardia, Propranolol, Qbrelis, Quinapril, Ramipril, Saluron, Sectral, Spironolactone, Sular, Tarka, Tartrate, Taztia, Tekturna, Telmisartan, Telmisartan Eprosartan, Tenex, Tenoretic, Tenormin, Terazosin, Teveten, Thalitone, Tiadylt, Tiazac, Timolol, Toprol, Toprol XL, Torsemide, Trandate, Trandolapril, Tribenzor, Twynsta, Univasc, Valsartan, Vaseretic, Vasotec, Vecamyl, Verapamil, Verelan, Zaroxolyn, Zebeta, Zestoretic, Zestril, Ziac |
| ICD-9 | 401, 402, 403, 404, 405 |
| ICD-10 | I10, I11, I12, I13, I14, I15 |

**Table 4.3. Comorbidity Inclusion Criteria – Hypercholesterolemia**

| **Code Description** | **Code(s) or Keyword(s)** |
| --- | --- |
| Diagnosis Keywords | Cholesterol, Dyslipidemia, Hyperlipidemia, Hypercholesterolemia, High Lipids, High Cholesterol, High LDL |
| Medication Keywords | Altoprev, Advicor, Alirocumab, Altocor, Amlodipine, Antara, Atorvastatin, B-3-50, B3-500-gr, Caduet, Cholestyramine, Colesevelam, Colestid, Colestipol, Crestor, Endur-acin, Evolocumab, Ezallor, Ezetimibe, Fenofibrate, Fenofibric Acid, Fenoglide, Fibricor, Flolipid, Fluvastatin, Gemfibrozil, HDL benefit, Juxtapid, Lescol, Lipitor, Lipofen, Livalo, LoCholest, Lomitapide, Lopid, Lovastatin, Lovaza, Mevacor, Niacin, Niacor, Niaspan, Nicotinic Acid, Omega-3 Acid Ethyl Esters, Pitavastatin, Praluent, Pravachol, Pravastatin, Prevalite, Questran, Repatha, Rosuvastatin, Simcor, Simvastatin, Tricor, Triglide, Trilipix, Vytorin, Welchol, Zetia, Zocor, Zypitamag |
| ICD-9 | 272.9, 272.4 |
| ICD-10 | E78.9, E78.5 |
